# Hydrogen-Bonded and Halogen-Bonded: Orthogonal Interactions for the Chloride Anion of a Pyrazolium Salt

**DOI:** 10.3390/molecules26133982

**Published:** 2021-06-29

**Authors:** Steven van Terwingen, Daniel Brüx, Ruimin Wang, Ulli Englert

**Affiliations:** 1 Institute of Inorganic Chemistry, RWTH Aachen University, Landoltweg 1, 52074 Aachen, Germany; steven.vanterwingen@ac.rwth-aachen.de (S.v.T.); daniel.bruex@rwth-aachen.de (D.B.); ruimin.wang@ac.rwth-aachen.de (R.W.); 2 Institute of Molecular Science, Shanxi University, Taiyuan 030006, China

**Keywords:** halogen bond, hydrogen bond, single-crystal XRD, DFT calculation, QTAIM

## Abstract

In the hydrochloride of a pyrazolyl-substituted acetylacetone, the chloride anion is hydrogen-bonded to the protonated pyrazolyl moiety. Equimolar co-crystallization with tetrafluorodiiodobenzene (TFDIB) leads to a supramolecular aggregate in which TFDIB is situated on a crystallographic center of inversion. The iodine atom in the asymmetric unit acts as halogen bond donor, and the chloride acceptor approaches the σ-hole of this TFDIB iodine subtending an almost linear halogen bond, with Cl···I = 3.1653(11) Å and Cl···I–C = 179.32(6)°. This contact is roughly orthogonal to the N–H···Cl hydrogen bond. An analysis of the electron density according to Bader’s Quantum Theory of Atoms in Molecules confirms bond critical points (bcps) for both short contacts, with $\rho_{\mathrm{bcp}}$ = 0.129 for the halogen and 0.321 *e*${Å}^{-3}$ for the hydrogen bond. Our halogen-bonded adduct represents the prototype for a future class of co-crystals with tunable electron density distribution about the σ-hole contact.

## 1. Introduction

Starting with his first publications in the 1920s, Linus Pauling took a never-ceasing interest in both theoretical [1,2] and experimental [3,4] insights in chemical bonds. This fascination is reflected by his famous textbook *The Nature of the Chemical Bond* [5] and peaked in him being awarded the Nobel prize in chemistry in 1954 “for his research into the nature of the chemical bond and its application to the elucidation of the structure of complex substances” [6]. Pauling achieved the aforementioned experimental elucidation of chemical structures predominantly by X-ray methods, e.g., single-crystal X-ray diffraction (SCXRD). In *The Nature of the Chemical Bond*, Pauling also recognized the strongly electrostatic nature of hydrogen bonds.

**Figure 1:**
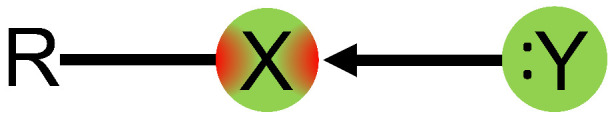
The halogen X exhibits an electron deficient site (red, δ+) in direction of the σ-bond to R. The nucleophile Y can interact with this positively charged region *via* its lone pair, thus forming a halogen bond.

A halogen’s ability to form favourable interactions with nucleophiles is called a *halogen bond* [7]. Similar to hydrogen bonds, they form between an electron donating and an electron accepting site; in the case of halogen bonds the halogen is the electron accepting partner (Figure 1) [7,8].

By definition, the halogen is the *donor* of the halogen bond. Because of the localization of the electron deficiency on the opposing site of the σ-bond to R it is called the σ*-hole* [9]. First crystallographic evidence for a halogen bond was given by the Hassel group in 1954: they described the 1:1 adduct of 1,4-dioxane with Br_2_ [10]. Although the term halogen bond was not used until introduced by Dumas et al. in 1978, this short O···Br contact of approx. 2.71 Å paved the way for forthcoming research regarding the character of these interactions. Since then, halogen bonds have been studied and utilized extensively in various fields including macromolecular and supramolecular chemistry, theory concerning chemical bonds and crystal engineering.

More recently, halogen bonds and related interactions have gained increasing attention from theory [11,12,13,14], and the σ-hole model has also been supported by experimental charge density studies [15,16,17,18,19]. In view of their occurrence in various fields, including macromolecular and supramolecular chemistry, halogen bonds have also been applied in the design of extended solids, i.e., in crystal engineering [20]. It has also been shown that molecules with enforced intramolecular hydrogen bonds to a halogen bond donor form stronger halogen bonds to adjacent halides [21].

**Figure 2:**
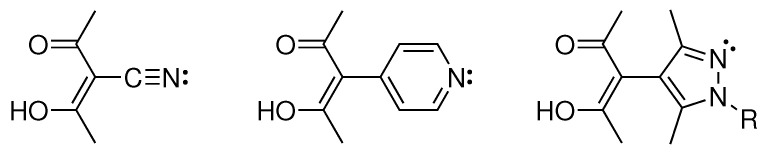
Chemical structures of heterobifunctional molecules utilized by our group for crystal engineering exhibiting nitrogen lone pairs.

For the design of extended structures, we often employ heterobifunctional molecules. Their different sites can selectively interact to form well-ordered assemblies, mostly based on coordinative bonds [22]. As our ditopic molecules usually exhibit functional groups with nitrogen lone pairs, e.g., nitriles [23,24], pyridines [25] or pyrazoles [26,27], they may alternatively engage as nucleophiles in halogen bonds (Figure 2) [28].

We have recently shown that pyrazolyl substituted acetylacetones are heterobifunctional molecules suitable for the construction of well-ordered bimetallic coordination polymers [27]. In this study we do not report metal coordination but rather hydrogen and halogen bonds with a hydrochloride. We discuss the synthesis and characterization of 3-(3,5-dimethyl-1-phenyl-1*H*-pyrazol-4-yl)acetylacetone (HacacPhPz, **1**) and the cocrystal formed by its hydrochloride with tetrafluorodiiodobenzene (TFDIB) **2**, a popular building block in crystal engineering [20].

## 2. Results and Discussion

### 2.1. Experimental Crystal Structures

**Figure 3:**
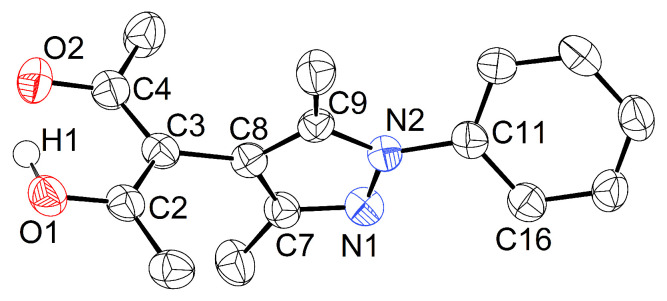
Displacement ellipsoid plot [30] of one molecule in the asymmetric residue of **1** (80% probability, C bonded hydrogens omitted). Selected intramolecular distances and angles (Å, °): O1–C2 1.311(2), O2–C4 1.278(2), C2–C3 1.395(3), C3–C4 1.424(3), C2–C3–C8–C9 85.7(2), N1–N2–C11–C16 28.6(2).

We will first address the heterobifunctional molecule HacacPhPz itself. Like our previous reported compounds, it is obtained by the reaction of the appropriate substituted hydrazine derivative, e.g., phenylhydrazine, with tetraacetylethane [26,27,29]. HacacPhPz (**1**) crystallizes in the orthorhombic space group Pbca with Z=16 and $Z^{\prime }=2$; one of the independent molecules is shown in Figure 3.

Like most acetylacetones substituted in 3-position, **1** predominantly exists as the enol tautomer in solid state as well as in solution (Figure S3).

**Figure 4:**
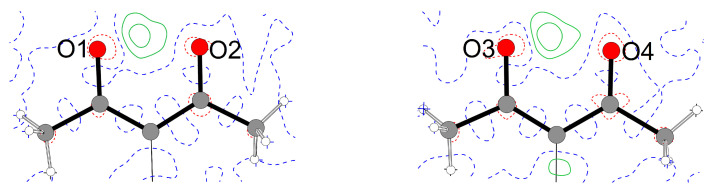
Difference Fourier contour map [30] of the acetylacetone moiety in both molecules contained in the asymmetric residue of **1**. Contour lines are drawn at 0.2 *e*${Å}^{-3}$ (red: positive difference, green: negative difference, blue: zero lines).

In the crystalline solid at 100 K, the enol hydrogen is clearly localized and is detected as local electron density maximum in a difference Fourier map (Figure 4). This assignment is corroborated by the C–O and C–C bond lengths in the acetylacetone moiety: in the case of the first molecule in the asymmetric residue depicted in Figure 4, O1–C2 (1.311(2) Å) is significantly longer than O2–C4 (1.278(2) Å), and in the carbon backbone C2–C3 (1.395(3) Å) is shorter than C3–C4 (1.424(3) Å).

In the following comparison, references are accompanied by their CCDC refcodes. Unsubstituted acetylacetone (refcode LIWPIQ [31]) crystallizes as the enol in the orthorhombic space group Pnma with the crystallographic mirror plane perpendicular to the least squares plane through the molecule, running through C3 and the hydrogen attached to it. For reasons of symmetry, disorder of the enol H with half occupancy is enforced. Boese et al. noted that a stable refinement of the structure could be conducted in the non-centrosymmetric subgroup $Pna2_{1}$, with two local electron density maxima for the disordered enol H; occupancies resulted as equal within error. The interatomic C–O (1.2909(13) Å) and C–C (1.4028(16) Å) distances in unsubstituted acetylacetone represent average values of the bond lengths found in **1**. The C=O bond distance for the keto group in **1** (1.278(2) Å) is slightly longer than corresponding distances in small ketones such as methyl ethyl ketone (MEK, LASLAU [32], 1.2128(19) Å) or pentanone (ZEJFEZ01 [33], 1.216(4) Å). In contrast, the C–C bond adjacent to the keto side in **1** (1.395(3) Å) is shorter than in the non-conjugated compounds (1.485(2) Å for LASLAU, 1.493(5) Å for ZEJFEZ01). We terminate our comparison with the literature by referring to a very recent article by Martinez et al. [34] in which the authors could correlate the enol H position, i.e., acetylacetone tautomerism with the presence of halogen bonds.

**Figure 5:**
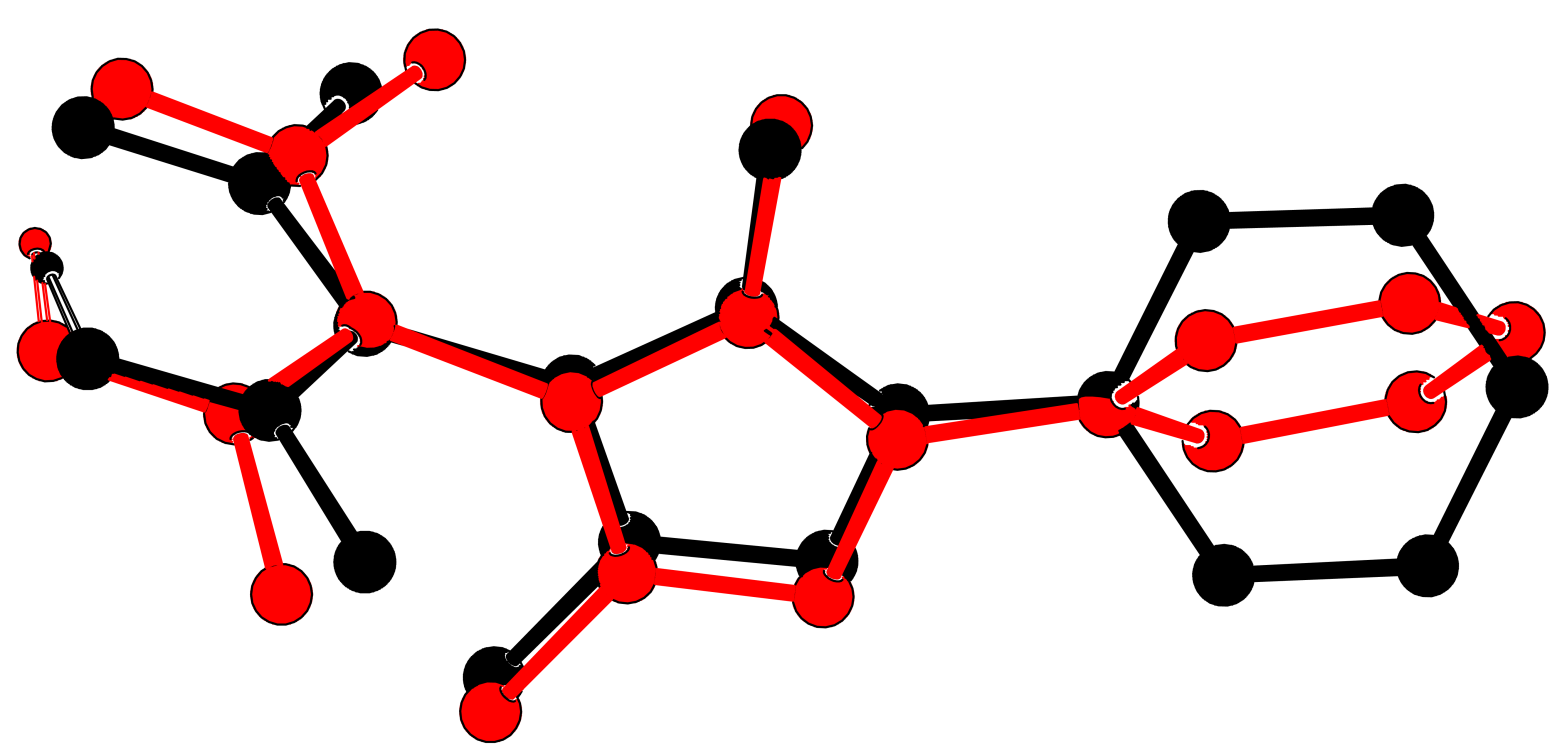
Overlay plot [30] of both molecules in the asymmetric residue in **1** (black: molecule 1 as shown in Figure 3, red: molecule 2 under symmetry operator −x,−y,−z; C bonded hydrogens omitted).

An overlay of both molecules in the asymmetric unit (Figure 5) reveals that the independent residues do not adopt the same conformation. Their planar subunits acetylacetone, pyrazolyl and phenyl subtend different dihedral angles.

In the packing in **1**, no particularly close directional interactions occur. Acetylacetone moieties and phenyl substituents aggregate in separate regions; C–H···O interactions of approx. 2.5 Å represent the shortest intermolecular contacts.

**Figure 6:**
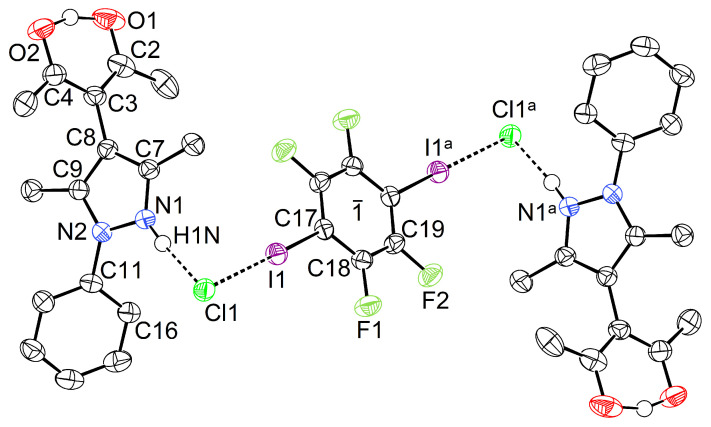
Displacement ellipsoid plot [30] of **2** (80% probability, C bonded hydrogens omitted). Selected intramolecular distances and angles (Å, °): I1···Cl1 3.1653(11), Cl1···N1 2.970(2), I1···Cl1···N1 73.99(4), C17–I1···Cl1 179.32(6), Cl1···H1N–N1 172(3). Symmetry operation: a = 1−x,−y,1−z.

In order to probe the influence of the N-substitution on the ability to form halogen bonds, co-crystallization of **1** with TFDIB was attempted. We have not been able to isolate any adduct, presumably due to steric congestion about the nucleophilic N1, but this site was still expected to be accessible by a proton. Therefore, hydrochloric acid was added and crystals of the solid HacacPhPz·HCl·0·5TFDIB (**2**) were obtained. This product crystallizes in the monoclinic space group $P2_{1}/c$ with Z=4, i.e., with a TFDIB moiety on a crystallographic centre of inversion (Figure 6).

The chloride anion Cl1 bridges two protonated H_2_acacPhPz^+^ cations to a central TFDIB moiety *via* two roughly orthogonal short contacts. The first interaction involves a classical N–H···Cl hydrogen bond, with a donor···acceptor distance of 2.97 Å; additional geometric details have been compiled in the caption of Figure 6. We recall that Pauling has emphasized the strongly electrostatic nature of hydrogen bonds as early as 1939. In the second short contact, the chloride ion acts as halogen bond acceptor: in agreement with σ-hole theory [7,8], it approaches the iodine atom in the direction opposite to the C–I bond; the arrangement Cl1···I1–C17 is almost linear. Only a few examples of this kind have been described to date [35,36,37,38,39,40], and Table 1 provides an overview of the most relevant geometry parameters.

Both linear and roughly orthogonal interactions are known for interhalogene ions. According to the popular VSEPR (Valence Shell Electron Pair Repulsion, aka Gillespie-Nyholm) interpretation [41,42] a linear disposition for two bonding partners and three lone pairs can be expected in formal 10 valence electron species such as the central iodine in I_3_^−^ or the recently described ClF_2_^−^ ions [43]. In contrast, for a formal 8 electron moiety such as ClF_2_^+^ [44] or all the examples given in Table 1 in which a Cl^−^ anion formally employs two out of four lone pairs, one for accepting the hydrogen bond and one as a nucleophile towards iodine, a bent arrangement can be predicted.

### 2.2. Theoretical Evaluation of the Halogen Bond

**Figure 7:**
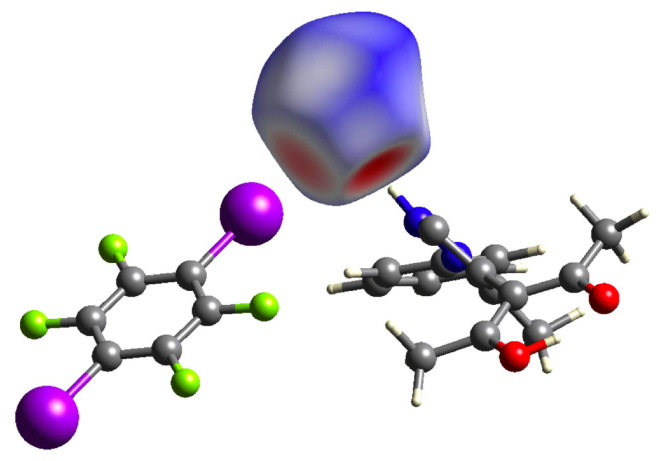
Hirshfeld surface [46] around Cl1 mapped with $d_{\mathrm{norm}}$; regions marked in red represent directions of short, those in blue of long contact distances.

The Hirshfeld surface [45] about the chlorine anion is shown in Figure 7 and clearly reflects the short contacts mentioned above as red contact regions.

**Figure 8:**
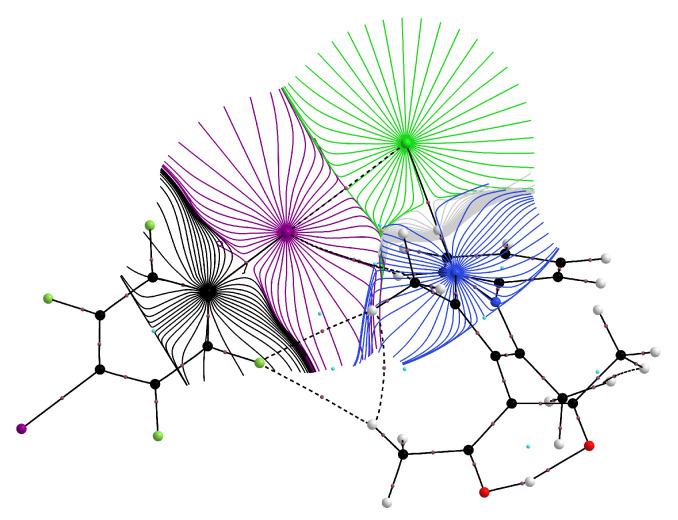
Atomic basins [47,48] in **2**; intramolecular bond paths and the conventional hydrogen bond are shown as solid black lines, the halogen bond and non-classical hydrogen bonds as dashed black lines.

Additional insight concerning the electron density distribution in **2** was obtained *via* a single point calculation, followed by an analysis of the resulting electron density by Bader’s Quantum Theory of Atoms in Molecules [47]. Figure 8 shows a trajectory plot of the gradient of the electron density from a similar view direction as the Hirshfeld surface in Figure 7; both the classical N–H···Cl hydrogen bond and the Cl···I halogen bond are associated with essentially linear bond paths and (3,−1) critical points (bcps).

Table 2 summarizes relevant characteristics of the electron density in the bcps of the short contacts and its derived properties.

We are not aware of experimental charge density studies on I···Cl halogen bonds and therefore cannot compare $\rho_{\mathrm{bcp}}$ for I1···Cl1 to other structures of the same class but we have encountered similar values for the shortest Cl···Cl contacts investigated by high resolution X-ray diffraction [49,50]. Geometric and electronic parameters of the classical N–H···Cl hydrogen bond may be compared to the situation in bis(2-chloropyridinium) tetrachloridozincate; for the latter compound, the electron density was established experimentally [51]. Table 2 shows that the calculated properties for the hydrogen bond in **2** and our previous experimental observations for a contact of similar geometry match well, albeit the experimental values are obviously associated with appreciable standard uncertainties. In addition to distance criteria and $\rho_{\mathrm{bcp}}$, energy densities in the bond critical point have proven useful to categorize short contacts. Only the hydrogen bond is associated with a negative total energy density *E*, the sum of the (positive) kinetic energy density *G* and the (negative) potential energy density *V*. The halogen bond, in contrast, is characterized by a small positive $E_{\mathrm{bcp}}$, typically encountered for weak closed shell interactions [52].

**Figure 9:**
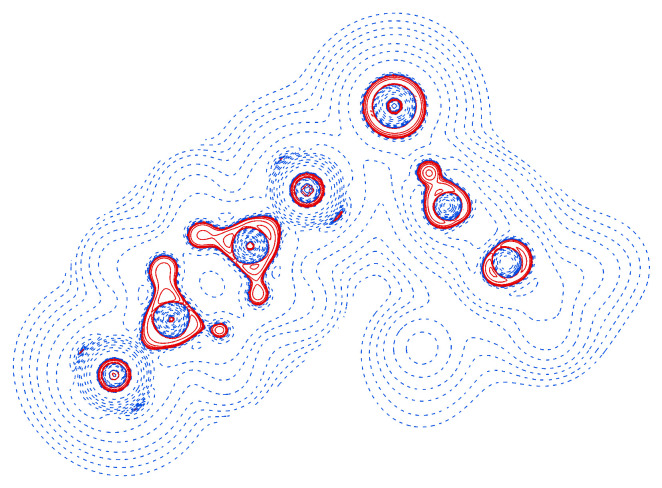
Laplacian of the electron density in **2**; positive values in blue, negative values in red, contours at $\pm 2^{n}\;\cdot \;10^{-3}$ a. u. (0≤n≤20).

The Laplacian of the electron density in the region of the short contacts is depicted in Figure 9. A slight polarization of the Cl^−^ anion towards the hydrogen can be perceived. The iodine atom is associated with two well visible valence charge concentrations perpendicular to the σ and the halogen bond.

**Figure 10:**
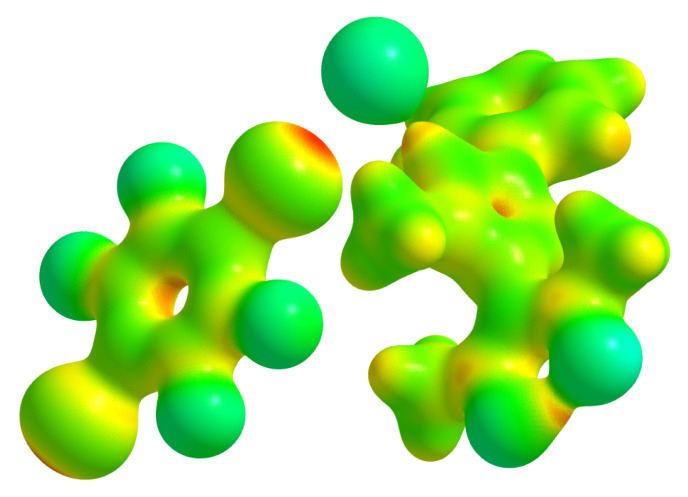
Electrostatic potential for **2**, mapped on an isosurface of electron density ρ = 0.05 a. u. [48]; red areas are associated with a positive value (0.480 a. u.), cyan areas with negative values (−0.0675 a. u.) and green areas with an ESP (0.115 a. u.).

The short contact between the chloride anion and the iodine atom of the fluorinated aromatic ring does not only reflect the geometry of a σ-hole interaction but also matches the electrostatics of such a contact: Figure 10 shows the electrostatic potential for **2**. The positive region on the iodine atom opposite to its covalent bond to the ring carbon atom is clearly visible.

## 3. Conclusions and Outlook

Hydrogen bonds and halogen bonds contribute to the structure of crystalline **2**. This balance does not come by accident and may become a useful motif in crystal engineering. Hydrochlorides and -bromides of a wide range of basic compounds are readily available. The interaction of their halide anion X^−^ with a halogen bond donor represents a rarely exploited way to tune and investigate both the geometry and the charge density of halogen bonds. The formation of a classical hydrogen bond between the protonated site and X^−^ will often occur, and the halide engaged in this interaction can be expected to act as a good halogen bond acceptor, both with respect to its electronic and steric properties. The possibility to vary the parent base, the hydrogen halide, and the halogen bond donor molecule makes this approach attractive for the systematic study of halogen bonds. In favorable cases, the crystalline products will not only allow conventional structural studies and theoretical calculations but can also become the target of experimental charge density studies.

## 4. Experimental Section

### 4.1. Computational Details

Prior to the single point calculation, C–H and N–H distances were idealized to values consistent with results from neutron diffraction [53]. The single point calculation was performed with Gaussian [54]; the MIDIX basis set [55] was used. In the calculation complete residues were taken into account and therefore a slightly expanded asymmetric unit was used. It comprised the residues depicted in Figure S5, i.e., a full rather than a half TFDIB molecule. The electron density associated with the single point calculation was analyzed with AIMAll [48] and Multiwfn [56] and interpreted according to Bader’s QTAIM [47]. The kinetic energy density *G* and the ratio between kinetic energy density and electron density, G/ρ in the bcp, were derived as suggested by Abramov [57], and the potential energy density *V* was obtained according to the local virial theorem [58,59].

### 4.2. Materials and Methods

All chemicals were used without further purification. Magnetic resonance spectra were measured using a Bruker Avance II UltrashieldT11 plus 400 instrument (400 MHz, referenced to SiMe_4_). Infrared spectra were recorded using a Nicolet Avatar 360 E.S.P. spectrometer in potassium bromide windows. Elemental analyses were performed using a Heraeus CHNO-Rapid VarioEL. 3,4-Diacetylhexane-2,5-dione (tetraacetylethane, TAE) was prepared according to known procedures [26,29,60]. Intensity data was collected with a Bruker D8 goniometer equipped with an APEX CCD area detector and an Incoatec microsource (Mo-K${}_{\alpha }$ radiation, λ = 0.71073 Å, multilayer optics). Temperature was maintained by using an Oxford Cryostream 700 instrument, Oxfordshire, UK. Data was integrated with SAINT [61] and corrected for absorption by multi-scan methods [62]. The structures were solved by intrinsic phasing [63] and refined by full matrix least squares procedures against $F^{2}$, as implemented in SHELXL-18. Crystal data, data collection parameters and refinement results have been compiled in Table S1. CIFs have been deposited under CCDC No. 2086575 (**1**) and 2086574 (**2**). Powder diffraction experiments were performed on flat samples at room temperature using a STOE STADI P diffractometer with Guinier geometry (Cu-K${}_{\alpha 1}$, λ = 1.54059 Å, Johann germanium monochromator, STOE image plate detector IP-PSD, 0.005° step width in 2θ).

### 4.3. Synthesis of 3-(3,5-Dimethyl-1-phenyl-1 *H*-pyrazol-4-yl)acetylacetone, HacacPhPz, ***1***

TAE (0.991 g, 5.0 mmol, 1.0 eq.) was dispersed in absolute ethanol (50 mL) under N_2_ atmosphere and heated to reflux. When all TAE was dissolved, phenylhydrazine (0.49 mL, 5.0 mmol, 1.0 eq.) was added dropwise and the solution was kept at reflux for an additional 5 h. The solvent was removed under reduced pressure yielding a yellow oil. Column chromatography (silica, *n*-pentane/EtOAc, 3:1, *v*:*v*; $R_{f}=0.71$) yields a pale yellow solid. Yield: 523 mg (39%). Colorless plate-shaped crystals suitable for SCXRD were obtained after recrystallization from *n*-hexane. ${}^{1}$H NMR (CDCl_3_, 400 MHz): δ 16.89 (s, 1H), 7.51–7.42 (m, 4H), 7.41–7.33 (m, 1H), 2.18 (s, 6H), 1.94 (s, 6H). ${}^{13}$C{${}^{1}$H} NMR (CDCl_3_, 100 MHz): δ 192.83, 148.73, 139.94, 138.16, 129.27, 127.63, 124.68, 115.34, 104.20, 23.87, 12.33, 11.52. CHN: anal. calcd. for C_16_H_18_N_2_O_2_: C: 71.1%, H: 6.7%, N: 10.4%; found: C: 70.6%, H: 6.6%, N: 10.2%. HRMS-ESI (m/z): [M + H]${}^{+}$ calcd. for C_16_H_19_N_2_O_2_^+^: 271.14410; found: 271.14409. mp: 63.5 ${}^{\circ }C$. While some similarities can be derived from the simulated and experimental powder pattern, phase purity could not be confirmed (Figure S1).

### 4.4. Synthesis of HacacPhPz·HCl·0·5TFDIB, ***2***

HacacPhPz (13.6 mg, 0.05 mmol, 2.0 eq.) and TFDIB (10.1 mg, 0.025 mmol, 1.0 eq.) were dissolved in CHCl_3_ (0.25 mL) each. The two solutions were combined and placed in a bigger vial containing aq. HCl (0.91 mL, 5.4 M) for slow vapor diffusion. Colorless crystals were obtained after slow evaporation of the solvent at room temperature. Yield: 13.4 mg (76%). The powder pattern of ground **2** does not match the simulation of the experimentally established single crystal structure (Figure S1, bottom). In order to investigate whether **2** decomposes upon grinding, a moist pH-indicator paper was placed in the head space of a bulk sample of **2**. The color change of this indicator within several hours proved the release of a gaseous acid, most likely HCl. We do, however, not observe complete loss of HCl and re-generation of the constituents **1** and TFDIB but formation of an unknown crystalline product (Figure S2). On the scale of single crystals rather than ground powder, the loss of HCl is slower, in agreement with the matching microanalytical data.

## Figures and Tables

**Table 1:**
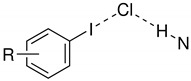
Comparison between important distances and angles in **2** and literature known compounds with a similar structure motif.

Compound	*d*(I···Cl)/Å	*d*(Cl···N)/Å	*∠*(C–I···Cl)/°	*∠*(I···Cl···N)/°
**2**	3.1653(11)	2.970(2)	179.32(6)	73.99(4)
BEXPOL [35]	3.211	3.085	177.81	111.72
JAQNAR [36]	3.331	3.137	169.49	92.01
RUWVUB ${}^{†}$ [37]	3.223	3.133	172.72	106.15
RUWWIQ ${}^{†}$ [37]	3.102	3.103	179.00	111.33
VIDHEY [38]	3.489	3.124	169.32	71.89
WOQRIF [39]	3.422	2.989	160.38	77.60
JULRIU [40]	3.122	3.003	176.41	90.99
JULSAN [40]	3.240	3.033	171.83	101.30

${}^{†}$ For multiple hits in the same structure their average value was calculated and is presented here.

**Table 2:** Short contacts with properties of their bond critical point (bcp) (3,−1) in **2** and experimental data from LAVNUU [49].

Bond	I1···Cl1 in 2	Cl1···H1N in 2	Cl···H in LAVNUU [49]
ρ/$e{Å}^{-3}$	0.129	0.321	0.28
$\nabla^{2}\rho$/$e{Å}^{-5}$	1.184	1.785	0.6
bond path length/Å	3.1654	2.0680	2.11
*G*/a. u.	0.0110	0.0300	0.018
G/ρ/a. u.	0.58	0.63	0.44
*V*/a. u.	−0.0097	−0.0415	−0.030
*E*/a. u.	0.0123	−0.0115	−0.012

## Data Availability

Data is contained within the article or Supplementary Materials.

## Supplementary Materials

The following are available online, Figure S1: Simulated and experimental powder patterns of **1** (top) and **2** (bottom), Figure S2: Experimental powder patterns of **2** and simulated patterns of **1** and TFDIB [64], Figure S3: ${}^{1}$H (top) and ${}^{13}$C{${}^{1}$H} (bottom) NMR spectra of **1** measured in CDCl_3_ (*) at room temperature, Figure S4: HSQC (red) and HMBC (green) NMR spectra of **1** measured in CDCl_3_ (*) at room temperature, Figure S5: Structure fragment used for the single point calculation discussed in the main text, Section 2.2, Table S1: Crystal data and refinement results for SCXRD data for **1** and **2** measured at T=100 K, Table S2: Topological properties of interactions at their bond critical point (3,−1) of **2**.

## Abbreviations

The following abbreviations are used in this manuscript:

bcp	bond critical point
HacacPhPz	3-(3,5-dimethyl-1-phenyl-1*H*-pyrazol-4-yl)acetylacetone
QTAIM	Quantum Theory of Atoms in Molecules
SCXRD	single-crystal X-ray diffraction
TAE	tetraacetylethane
TFDIB	tetrafluorodiiodobenzene
